# Hydrochlorothiazide vs Venlafaxine: Drug-induced Bullous Pemphigoid

**DOI:** 10.7759/cureus.4999

**Published:** 2019-06-25

**Authors:** Srikanth Naramala, Hussain Dalal, Sreedhar Adapa, Pallav Patel, Venu Madhav Konala

**Affiliations:** 1 Rheumatology, Adventist Medical Center, Hanford, USA; 2 Internal Medicine, Internal Medicine Multi-Specialty Clinic, Houston, USA; 3 Nephrology, The Nephrology Group, Visalia, USA; 4 Internal Medicine, Kaweah Delta Medical Center, Visalia, USA; 5 Internal Medicine/ Hematology and Oncology, Ashland Bellefonte Cancer Center, Ashland, USA

**Keywords:** pemphigus, bullous pemphigoid, hydrochlorothiazide, venlafaxine

## Abstract

Pemphigoid group of dermatologic conditions is a group of autoimmune skin disorders resulting in blistering skin conditions. The two diseases that fall under this category are bullous pemphigoid and pemphigus vulgaris. While there are many similarities in these two disorders, there are numerous pathologic and biochemical differences which help us differentiate between these disorders. In this case report, we report a usual manifestation of bullous pemphigoid in a 72-year-old female secondary to use of a well-known antihypertensive (hydrochlorothiazide) and/or venlafaxine (anti-depressant).

## Introduction

In pemphigoid diseases of the skin, bullous pemphigoid is an autoimmune disorder most commonly found in older individuals. The disease predominantly affects patients over the age of 60. In the United States, approximately six to 13 cases per million patients are diagnosed every year, with equal incidence in both genders and no racial biases [1]. As described above, this is an autoimmune phenomenon where we find the presence of autoantibodies at the dermal-epidermal junction. Although it is an autoimmune disorder, this disorder can also be caused by the use of systemic drugs, such as certain diuretics, nonsteroidal anti-inflammatory drugs (NSAIDs), amoxicillin, gliptins and certain biologic agents [2]. As with any drug-induced reactions, discontinuation of the offending agent is the mainstay of the treatment. After an extensive review of literature, we report of a drug-induced bullous reaction as a side effect of one of the most commonly used anti-hypertensive, hydrochlorothiazide and along with venlafaxine.

## Case presentation

**Figure 1:**
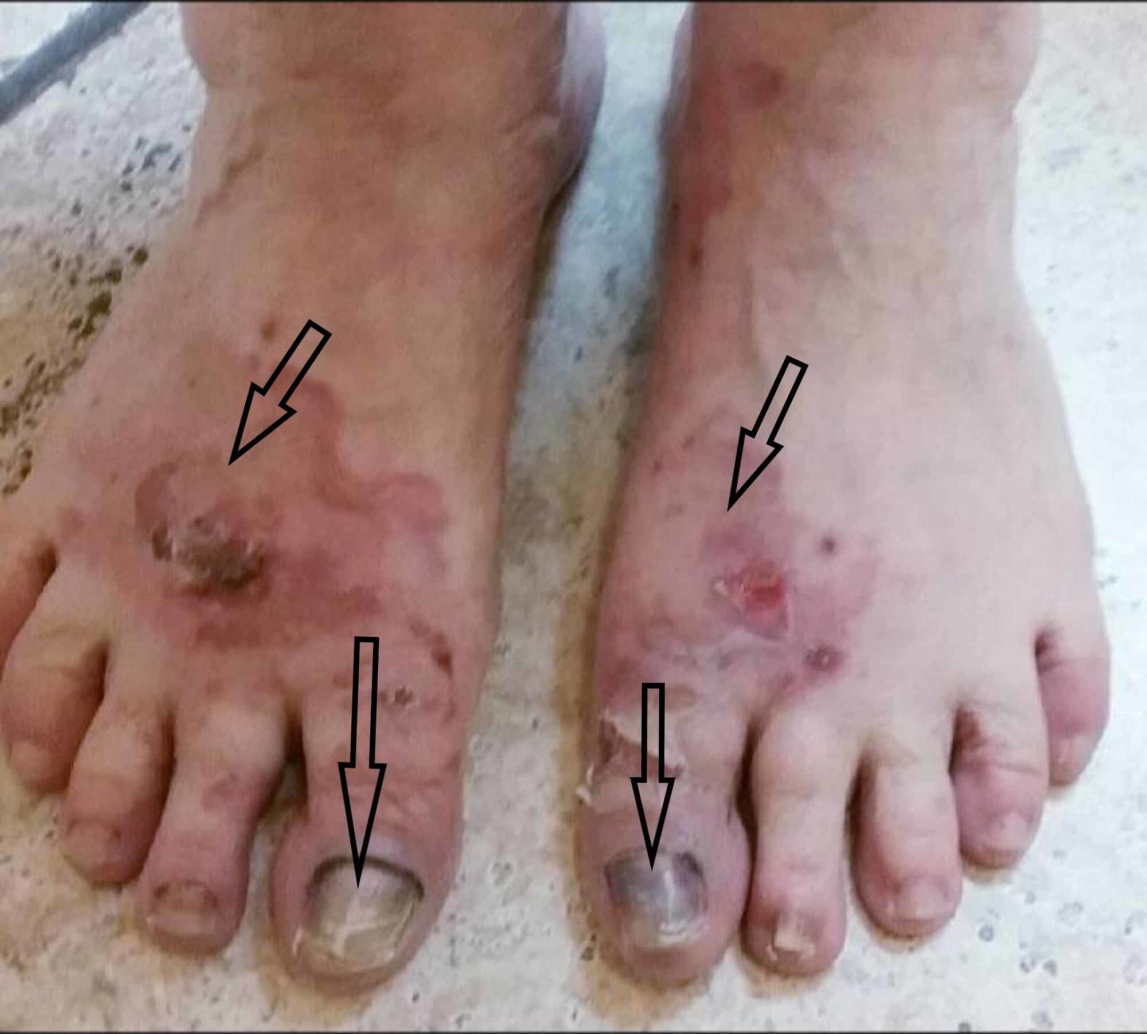
Ruptured bullae with open wounds on the bilateral feet, nail dystrophy with hyperpigmentation.

**Figure 2:**
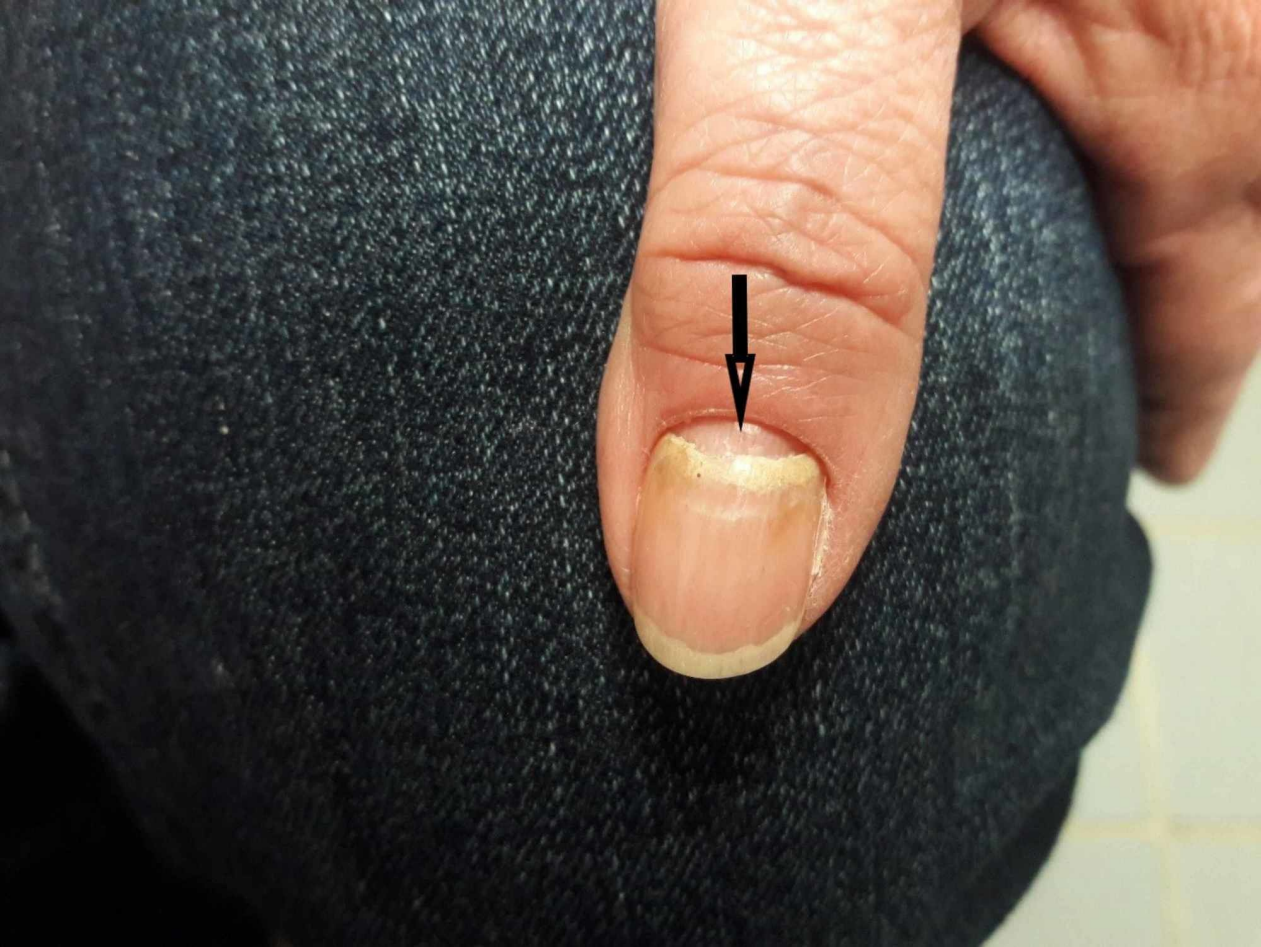
Onychomadesis of the nails - shedding of the nails beginning at the proximal end.

A 72-year-old Caucasian female with a past medical history of squamous cell cancer of the skin, hypothyroidism on Synthroid, and hypertension well-controlled with hydrochlorothiazide for the last seven years, was evaluated in rheumatology clinic for oral ulcers. She denies any recent travel, insect bites, any exposure to sick contacts, and similar complains in the past. Physical examination was significant for erythematous, ruptured bullae with crusted ulcerated lesion on the dorsal aspect of bilateral foot (Figure 1), scattered maculopapular lesions on bilateral legs, a single bullous lesion in left axilla, several bullous lesions in the oral cavity, significant hair loss along with nail changes in fingers and toes (Figures 1, 2).

She was initially evaluated by dermatology five months prior to presentation at the rheumatology clinic for a rash which started on bilateral thighs, later spreading to all over the body. A skin biopsy of the sub-clavicular region was done which showed nonspecific urticarial reaction with eosinophilic infiltrates which was thought to be secondary to a drug reaction. Direct immunofluorescence (DIF) assay was not performed on skin biopsy. Six weeks prior to the patient noticing the rash, she was started on venlafaxine 75 mg to overcome depression caused by her husband’s death. The venlafaxine and hydrochlorothiazide were stopped, but the patient’s rash did not improve. Over the next few months, the patient’s rash progressively worsened; she went to the emergency multiple times and was discharged on a brief course of antihistamines and oral steroids with minimal improvement.

She started noticing sores in her oral mucosa and was evaluated by ENT who referred the patient to rheumatology for evaluation of the autoimmune process. Review of blood work showed moderate eosinophilia with absolute eosinophil count at 1169/ul (18-500 cells/ul), which was still persistent but decreased on repeat labs with an absolute eosinophil count of 722 cells/ul (15-500 cells/ul). Extensive autoimmune serologies with antinuclear antibody, immunofluorescence assay, rheumatoid factor, antineutrophilic cytoplasmic antibody along with myeloperoxidase and proteinase-3 antibody, immunoglobulins A, G, M, and E, complements and bullous pemphigoid antibodies (BP 180 and BP 230) were negative. Infectious workups including HIV, hepatitis B core antibody, hepatitis B surface antigen, hep C antibody, rapid plasma reagin, and parvovirus IgM were also negative. The patient was then referred to dermatology for additional biopsy with special instructions to do DIF on the specimen. DIF of the right foot revealed linear deposits of C3 along the junctional zone and negative immunoglobulins, consistent with bullous pemphigoid. Blood work was positive for desmoglein-3 antibody at 161 u/ml (<9 u/ml), negative for desmoglein-1 antibody. She also had age-matched screening workup including mammogram, pap smear, and colonoscopy which were negative.

**Figure 3:**
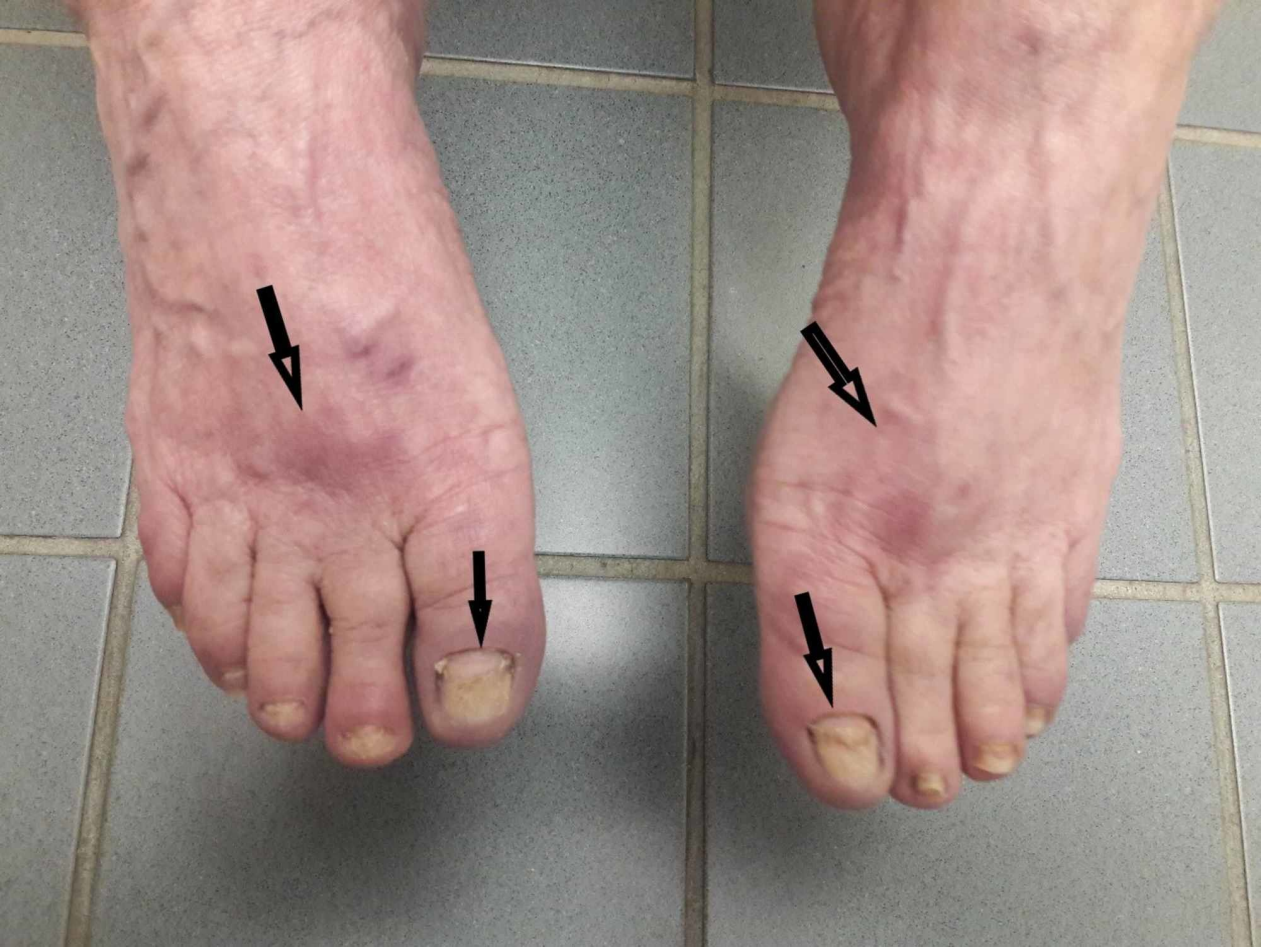
Four weeks post treatment with oral prednisone - Complete resolution of bullae and open wounds, significant improvement of nail changes.

**Figure 4:**
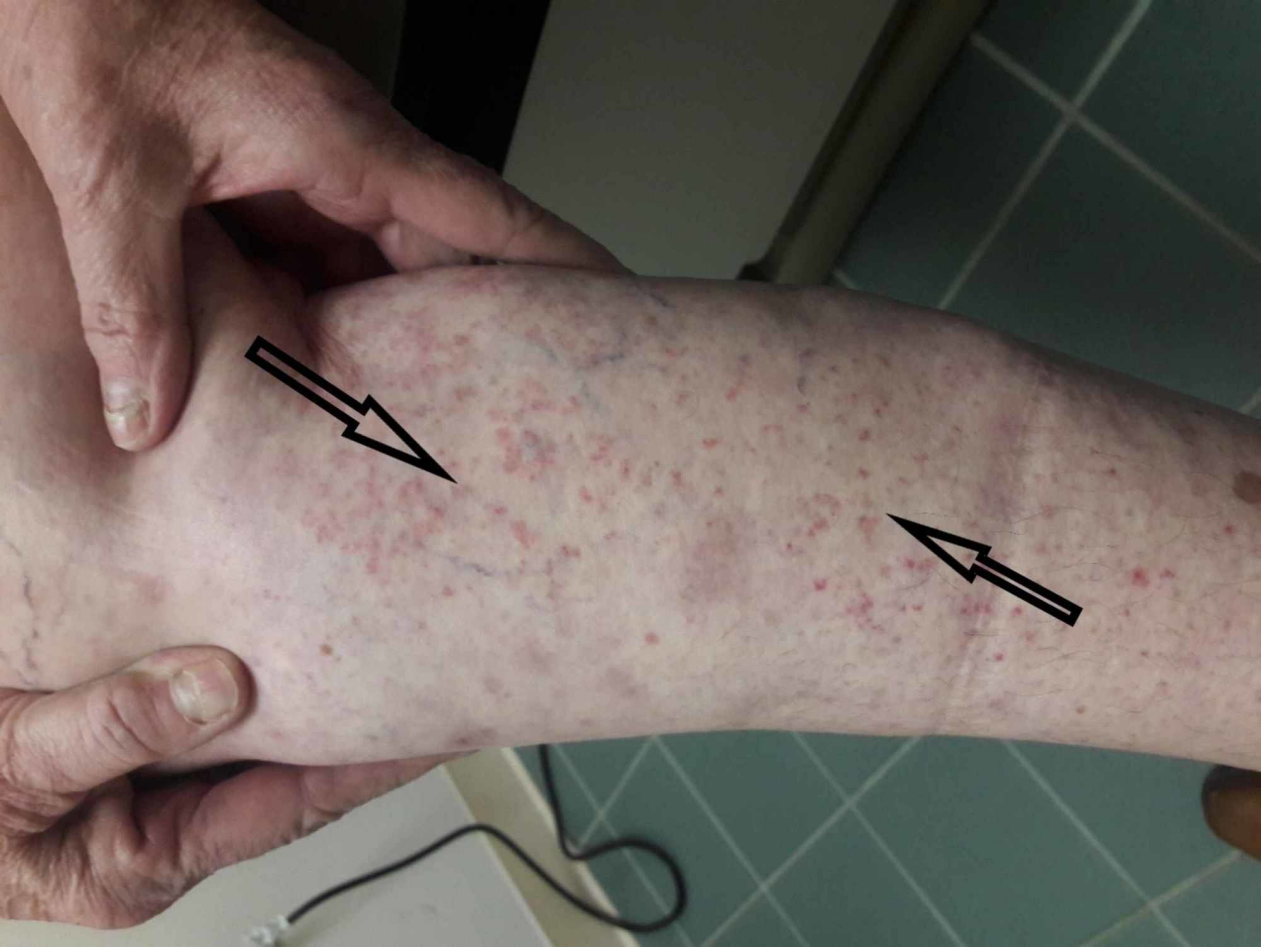
Recurrence of rash on 5 mg prednisone.

The patient was started on prednisone 40 mg per day with tapper with the resolution of rash and oral lesions (Figure 3). Her rash and oral lesions recurred on 5 mg per day of prednisone (Figure 4). Her prednisone dose was increased back to 40 mg per day with a taper plan along with starting Imuran 100 mg per day. Her prednisone was tapered down and stopped in two months. Her disease is under control on Imuran 100 mg per day for the last three months.

## Discussion

Bullous pemphigoid is an immune-mediated blistering skin disorder characterized by subepidermal blisters and compromising 80% of cases in pemphigoid group of disorders [1]. It is characterized by subepithelial blister formation, deposition of immunoglobulins, and complements at the dermo-epidermal junction and mucosal basement membrane [3]. There is subsequent damage to the epithelial basement membrane. The binding of these antibodies to the epithelial basement membrane initiates a series of reactions resulting in separation of the epidermis and the dermis in the skin (giving rise to the classic bullae) as well as epithelium from subepithelial tissue in mucous membranes [3]. Typical findings in Bullous pemphigoid include tense, thick fluid-filled bullae on abdomen, groin, and extremities compared to bullae of pemphigus vulgaris which are thinner and can be smudged off with pressure. While the majority of the cases in pemphigoid group of disorders are immune-mediated, the drug-induced bullous disease should also be given important consideration.

Review of the literature shows that there are about 50 different medications that cause drug-induced bullous pemphigoid, and there are likely more medications as new therapies become available [2]. One of the notable medications from the class of diuretics is hydrochlorothiazide. There have been a handful of case reports in the literature linking hydrochlorothiazide use to Bullous pemphigoid disorder, noting that onset of clinical features in most cases occurring in as little as six weeks after initiation of therapy. In only one case report published by Garcia Sanchez et al., the onset of symptoms was “several years” after the initiation of hydrochlorothiazide, but it was unclear about the exact duration of HCTZ therapy in the patient [4].

Based on the Naranjo scoring system for adverse drug reaction, we propose a high probability of this patient’s Bullous pemphigoid reaction to hydrochlorothiazide and venlafaxine [5]. Hydrochlorothiazide takes higher probability as several cases in the literature have linked HCTZ therapy to bullous pemphigoid reaction, but venlafaxine is also a likelihood given that the patient was just started on this anti-depressant six weeks before the reaction. While there is no documentation in literature with venlafaxine-induced bullous pemphigoid, given the timeline of events and the scoring probability of this adverse drug reaction, venlafaxine could also be considered a causative agent.

Skin biopsy is most definitive in diagnosing bullous pemphigoid. Serological testing to detect bullous pemphigoid antibodies can be useful in supporting the diagnosis. Bullous pemphigoid is usually diagnosed with increased levels of bullous pemphigoid antigen 180 (BP180) and bullous pemphigoid antigen 230 (BP230) [3]. In a retrospective study by Roussel et al. the sensitivity and specificity of detecting combined BP180 and BP230 antibodies by ELISA was 87% and 88%, respectively [6]. However, in our patient, both the levels of BP 180 and BP 230 were normal. On the other hand, the level of desmoglein-3 antibody was elevated, with the level of desmoglein-1 antibody being in normal limits. Both these antibodies are usually elevated in patients with pemphigus vulgaris. Although this is rare, there have been case reports, for example, Sami et al. reporting of a presence of elevated desmoglein-3 antibody in patients who have been diagnosed histopathologically as Bullous pemphigoid [7].

Based on the clinical presentation and the findings of skin biopsy, we made a diagnosis of Bullous pemphigoid in our patient, possibly induced by hydrochlorothiazide and/or venlafaxine use.

## Conclusions

Bullous pemphigoid is the most common form of pemphigoid disorders, usually diagnosed in patients over the age of 60. It is an autoimmune phenomenon, which in some cases can be triggered by medication use. There are over 50 different types of medications which are known to cause Bullous pemphigoid. The use of hydrochlorothiazide and/or venlafaxine can also be a contributing factor in inducing a similar reaction. As with most drug-induced reactions, cessation of the offending agent tends to be the mainstay of treatment. Additional immunosuppressive treatment should be considered in refractory cases similar to our patient.
